# Skeletal, cardiac, and respiratory muscle function and histopathology in the *P448Lneo−* mouse model of FKRP-deficient muscular dystrophy

**DOI:** 10.1186/s13395-018-0158-x

**Published:** 2018-04-06

**Authors:** Qing Yu, Melissa Morales, Ning Li, Alexander G. Fritz, Ren Ruobing, Anthony Blaeser, Ershia Francois, Qi-Long Lu, Kanneboyina Nagaraju, Christopher F. Spurney

**Affiliations:** 1 Center for Genetic Medicine Research, Children’s Research Institute, Children’s National Health System, Washington, DC USA; 2 School of Pharmacy and Pharmaceutical Sciences, Binghamton University, State University of New York, Binghamton, NY USA; 3 Department of Oncology, Ruijing Hospital, School of Medicine, Shanghai Jiao Tong University, Shanghai, China; 4 McColl-Lockwood Laboratory for Muscular Dystrophy Research, Department of Neurology, Carolinas Healthcare System, Charlotte, NC USA; 5 Children’s National Heart Institute, Center for Genetic Medicine Research, Children’s National Health System, Washington, DC USA

**Keywords:** Limb-girdle muscular dystrophy, Congenital muscular dystrophy, Fukutin related protein (FKRP), *P448Lneo−* mice, Echocardiography, Plethysmography, Preclinical trials

## Abstract

**Background:**

Fukutin-related protein (FKRP) mutations are the most common cause of dystroglycanopathies known to cause both limb girdle and congenital muscular dystrophy. The *P448Lneo−* mouse model has a knock-in mutation in the FKRP gene and develops skeletal, respiratory, and cardiac muscle disease.

**Methods:**

We studied the natural history of the *P448Lneo−* mouse model over 9 months and the effects of twice weekly treadmill running. Forelimb and hindlimb grip strength (Columbus Instruments) and overall activity (Omnitech Electronics) assessed skeletal muscle function. Echocardiography was performed using VisualSonics Vevo 770 (FujiFilm VisualSonics). Plethysmography was performed using whole body system (ADInstruments). Histological evaluations included quantification of inflammation, fibrosis, central nucleation, and fiber size variation.

**Results:**

*P448Lneo−* mice had significantly increased normalized tissue weights compared to controls at 9 months of age for the heart, gastrocnemius, soleus, tibialis anterior, quadriceps, and triceps. There were no significant differences seen in forelimb or hindlimb grip strength or activity monitoring in *P448Lneo− *mice with or without exercise compared to controls. Skeletal muscles demonstrated increased inflammation, fibrosis, central nucleation, and variation in fiber size compared to controls (*p* < 0.05) and worsened with exercise. Plethysmography showed significant differences in respiratory rates and decreased tidal and minute volumes in *P448Lneo−* mice (*p* < 0.01). There was increased fibrosis in the diaphragm compared to controls (*p* < 0.01). Echocardiography demonstrated decreased systolic function in 9-month-old mutant mice (*p* < 0.01). There was increased myocardial wall thickness and mass (*p* < 0.001) with increased fibrosis in 9-month-old *P448Lneo−* mice compared to controls (*p* < 0.05). mRNA expression for natriuretic peptide type A (Nppa) was significantly increased in *P448Lneo−* mice compared to controls at 6 months (*p* < 0.05) and for natriuretic peptide type B (Nppb) at 6 and 9 months of age (*p* < 0.05).

**Conclusions:**

FKRP-deficient *P448Lneo−* mice demonstrate significant deficits in cardiac and respiratory functions compared to control mice, and this is associated with increased inflammation and fibrosis. This study provides new functional outcome measures for preclinical trials of FKRP-related muscular dystrophies.

**Electronic supplementary material:**

The online version of this article (10.1186/s13395-018-0158-x) contains supplementary material, which is available to authorized users.

## Background

Muscular dystrophies are a heterogeneous group of disorders characterized by progressive muscle weakness and can also affect the respiratory, cardiac, and central nervous systems. The clinical phenotype and prognosis vary significantly making the diagnosis and treatment different for each disease. However, the continued identification of specific genetic causes for the more common muscular dystrophies has led to a better understanding of disease pathogenesis and new therapeutic strategies [1].

One pathogenic mechanism of muscular dystrophies lies in the disruption of the dystrophin-glycoprotein complex (DGC) [2]. The DGC is responsible for linking the sarcolemmal membrane with the extracellular matrix (ECM) and transmitting contraction forces to maintain muscle cell membrane integrity [3]. The DGC is composed of multiple proteins including dystrophin, dystroglycans, multiple sarcoglycans, dystrobrevin, laminin, and collagens. Without even one of these proteins, membranes can tear and activate multiple pathogenic pathways that lead to cell death.

Alpha-dystroglycan (α-DG) is one component of the DCG and interacts with proteins in the ECM including laminin, perlecan, agrin, neurexin using glycosylated O-mannose sugar moieties [4]. Defective glycosylation of α-DG is the pathogenic basis of several muscular dystrophy subtypes known as dystroglycanopathies, including limb-girdle muscular dystrophy (LGMD) and congenital muscular dystrophy (CMD). These subtypes demonstrate heterogeneous phenotypes that can range from early presentations with severe eye and brain disease to more mild skeletal muscle disease in older patients. More than 17 genes are involved in the pathogenesis including *POMT1*, *POMT2*, *POMGnT*, *FKRP*, *Fukutin*, and *LARGE* acting as glycosyl-transferases in the O-mannosylation of α-DG [5]. The severity of disease is thought to be related to the effect of each mutation on degree of glycosylation and laminin binding ability [6]. *FKRP* is a gene that encodes fukutin-related protein and its mutations cause dystroglycanopathies of both LGMD and CMD phenotypes as well as muscle-eye-brain and Walker-Warburg syndrome [7–11]. FKRP has recently been demonstrated as a ribitol 5-phosphate transferase in the synthesis pathway of laminin binding glycan of α-DG [12].

Multiple mouse models were developed to study the role of FKRP and experimental therapies. These models show a range of phenotypes consistent with human FKRP diseases. In general, the severity of the reported mouse models follows the same trend as the severity observed in patients with the same mutations. Ackroyd et al. (2009) developed the model FKRP-Neo^Tyr307Asn^ that demonstrated reduced levels of FKRP transcript. However, the mutant mice died soon after birth and were therefore not useful for experimental therapy development [13]. Mouse models with the common mutation L276I were created by several groups [14–16]. However, dystrophic phenotype of the mutant mice is very mild with clearly observable pathology only after 6 months of age without significant involvement of respiratory and cardiac muscles, apparently milder than phenotype in patients with the same homozygous mutations. Another reported FKRP mutant mouse model contains P448L mutation associated with congenital muscular dystrophy type 1C (MDC1C) in clinic [8, 17]. The knock-in FKRP P448L (with neo cassette removed, referred as *P448Lneo−* mouse) homozygous mouse has been reported with a severe phenotype consistent with severe LGMD2I, but milder than MDC1C as almost all newborn mice survive and have a life-span of more than 1 year with near normal breeding capacity [18]. Also important, the mouse was reported to show involvement of respiratory and cardiac muscles with progressive fibrosis [16]. Blaeser et al. (2016) examined the *P448Lneo−* mouse and demonstrated increased diaphragmatic fibrosis and decreased cardiac function by 12 months of age [19]. This pattern of phenotype represents well the clinic manifestation of dystroglycanopathies, as a proportion of the patient population is associated with pulmonary and cardiac disease. Clinically, Pane et al. (2012) described cardiac involvement in 6% and pulmonary involvement in 12% of patients with congenital muscular dystrophies [20]. And significant cardiac disease can be seen in LGMD 2I, even leading to cardiac transplantation [21–23]. More recently, a study by Maricelli et al. also demonstrated cardiac dysfunction with and without exercise [24]. We consider the *P448Lneo−* mouse highly relevant and valuable for developing experimental therapies to FKRP dystroglycanopathy. Therefore, validation of the skeletal muscle phenotype and further characterization of respiratory and cardiac muscle are essential.

## Methods

### Animal care

This study was carried out in strict accordance with the recommendations in the Guide for the Care and Use of Laboratory Animals of the National Institutes of Health. All experiments were performed in accordance with Children’s National Health System IACUC approved protocol #30432. *P448Lneo−* homozygous male mice were generated in McColl Lockwood Laboratory (Charlotte, NC) and rederived and imported from Jackson Laboratory (Bar Harbor, ME) [18]. Age-matched male *C57BL/6J* (referred to as control, *C57*, or *BL6*) mice were purchased from Jackson Laboratory. Animals were ear tagged prior to group assignment and were housed in cages of standard dimension on ground corn cob bedding mixed with a soft recycled shredded paper (nesting material) called Tek Fresh. The animals were housed in a temperature controlled (20–24 °C) colony room with a 12-h light/dark cycle and received mouse chow and water ad libitum. No animals were euthanized prior to reaching end of study criteria.

## Experimental procedure

Groups A, B, and D were composed of *P448Lneo−* (*n* = 8) and control (*n* = 8) mice. Group C was composed of *P448Lneo−* (*n* = 8), exercised *P448Lneo−* (*n* = 12), and control (n = 8) mice. Group A was studied at 1 month of age when the skeletal muscle pathology becomes detectable. Group B was studied at 2 months of age. Group C was studied every month until 6 months of age. A 1-month interval was chosen with the aim to identify the peak of muscle degeneration and severity as the disease progresses. A separate group of *P448Lneo−* mice underwent exercise treadmill running until 6 months of age. Control mice did not undergo exercise testing. Group D was studied at 9 months of age when both histological and functional data have already shown severe and detectable defects. Table 1 shows the timing of different testing for each group.

**Table 1:** Timeline of experimental procedures performed on groups A, B, C, D of *P448Lneo−* and control mice. Groups A, B, and D were composed of *P448Lneo−* (*n* = 8) and control (*n* = 8) mice. Group C was composed of *P448Lneo−* (*n* = 8), exercised *P448Lneo−* (*n* = 12), and control (*n* = 8) mice. Group A was studied at 1 month of age. Group B was studied at 2 months of age. Group C was studied every month until age 6 months of age. A group of *P448Lneo−* mice underwent exercise treadmill running until 6 months of age. Control mice did not undergo exercise testing. Group D was studied until 9 months of age

Group	A	B	C						D
Timeline (months of age)	1	2	1	2	3	4	5	6	9
Treadmill exercise (2×/week only P448Lneo*−* mice)			x	x	x	x	x	x	
Body weight	x	x	x	x	x	x	x	x	x
Grip strength test			x	x	x	x	x	x	x
Digiscan activity			x	x	x	x	x	x	x
Echocardiography				x				x	x
Plethysmography				x				x	x
Serum creatinine kinase	x	x						x	x
Histology	x	x						x	x
Fibrosis	x	x						x	x

### Treadmill

The mice were placed on the treadmill (Columbus Instruments, Columbus, OH) twice a week, one per lane for 30 min running at 12 m per minute speed per TREAT-NMD SOP for chronic exercise protocol in dystrophic mice (http://www.treat-nmd.eu/research/preclinical/dmd-sops/). If a mouse rested at the end of the lane, the animal would be gently pushed back onto the treadmill surface to restart running. The treadmill tests started on mice at approximately 1 month of age and continued until 6 months of age. During the weeks of measurements including grip strength, activity monitor, echo, and plethysmography, treadmill running was avoided.

### Grip strength

Forelimb grip strength was measured by a grip strength meter (Columbus Instruments, Columbus, OH). The animal was held so that only the forelimb paws grasped the specially designed mouse flat mesh assembly and the mouse was pulled back until their grip was broken. The force transducer retained the peak force reached when the animal’s grip was broken, and this was recorded from a digital display. For hindlimb strength, an angled mesh assembly was used. Mice were allowed to rest on the angled mesh assembly, facing away from the meter with its hindlimbs at least one-half of the way down the length of the mesh. The mouse tail was pulled directly toward the meter and parallel to the mesh assembly. During this procedure, the mice resist by grasping the mesh with all four limbs. Pulling toward the meter was continued until the hindlimbs released from the mesh assembly. Five successful hindlimb and forelimb strength measurements within 2 min were recorded. The maximum values were used for analysis. The grip strength measurements were collected in the morning hours over a 5-day period. The mice were trained on the grip strength meter before the trial [25]. Forelimb and hindlimb maximal muscle strength were obtained as values of KGF (kilogram-force) and normalized to bodyweights as “KGF/kg.”

### Locomotor activity

Locomotor activity was measured using an open-field digiscan apparatus (Omnitech Electronics, Columbus, OH). Total distance, horizontal activity, and vertical activity were recorded every 10 min for 1 h as described previously [26, 27]. As with the grip strength, the activity data were collected in the morning hours over a 4-day period and the mice were trained in the open field apparatus prior to the trial [25].

### Echocardiography

Echocardiography was performed and quantitative measurements were made offline using analytic software (FujiFilm VisualSonics, Toronto, Ontario, Canada) as previously described [25]. Measurements included vessel diameters, ventricular chamber size, and blood flow velocities and timing across the atrioventricular and semilunar valves. M-mode images were used to measure left ventricular (LV) chamber sizes and wall thicknesses. Percent shortening fraction (SF) and ejection fraction (EF) were calculated from M-mode measurements. Myocardial performance index (MPI) was also calculated from Doppler measurements.

### Plethysmography

The whole body plethysmography system (ADInstruments, St. Paul, MN) utilized a custom mouse chamber developed by the Research Instrument Shop at the University of Pennsylvania to minimize dead space. Other components in the system included the spirometer (ML141), respiratory flow head (MLTL1), and the PowerLab 4/30 with LabChart software. The mouse was brought to the measurement room 15 min before the start of the measurement session to recover from the transportation and new environment stresses. The spirometer was calibrated every time the hardware was powered on to read in terms of flow (ml/s) rather than pressure (mv).Calibration of the plethysmography was performed with 1 ml of air injected into the animal chamber to correlate the injected volume (ml) with the differential pressure (mv) measured in the chamber by integration. A 700 ml/min flow of dry air through the chambers was constantly delivered to avoid CO_2_ and water accumulation and to maintain a constant temperature. The mouse was weighed and placed into the chamber first to acclimate for 15 min then the respiratory flow data was recorded for 10 min. For data analysis, values for respiratory rate, tidal volume (TV), minute ventilation (MV), TV normalized by body weight (TV/BW), and MV normalized by body weight (MV/BW) were recorded using LabChart software.

### Blood collection

Blood samples were taken via retro-orbital bleeding when the animals were euthanized and the serum collected was used for creatinine kinase levels.

### Tissue collection and histological evaluations

Animals were sacrificed via inhaled carbon dioxide and cervical dislocation, and tissue samples were obtained. All tissue samples were weighed using the Mettler ToLedo scale (Columbus, OH) prior to processing. Skeletal muscles (gastrocnemius, tibialis anterior, soleus, triceps, and quadriceps) from one side of the animal, half the diaphragm, half the heart, and whole brain were stored in formalin. The contralateral or other half of muscles were snapped frozen in isopentane cooled in liquid nitrogen and stored at − 80 °C for further analysis. Slides were prepared and stained by Histoserv Inc. (Gaithersburg, MD)**.** Histological evaluations were performed in a blinded manner using coded slides. One transverse tissue section per muscle per animal was analyzed. Whole muscle digital images of the tissues were taken at × 20 using NanoZoomer slide scanner (Hamamatsu Inc., Bridgewater, NJ) and were opened using NDP.view2 software. Each tissue section was analyzed throughout its entire area. The total number of inflammation foci (an interstitial group of 10 smaller inflammatory cell dark blue nuclei in a high-power field) was quantified. The entire tissue section area was measured (mm^2^), and all counts were normalized to the tissue area. The parameters including percentage of fibers with central nucleation and fiber diameter were measured using MetaMorph Microscopy Automation and Image Analysis Software on paraffin sections of gastrocnemius, triceps, quadriceps, and diaphragm.

### Quantification of fibrosis

Paraffin sections of gastrocnemius, diaphragm, and heart tissue were stained with picrosirius red by Histoserv, Inc. (Germantown, MD). The tissues were magnified under a light microscope at an objective of 10 x and digital images obtained using computer software (Olympus C.A.S.T. Stereology System, Olympus America Inc., Center Valley, PA). These digital images were processed using ImageJ (NIH) with additional threshold color plug-ins to process jpeg images. Pixels corresponding to the area stained in red were normalized to the total pixel area of the tissue image, and the results were expressed as percent of fibrotic area.

### mRNA expression analysis

Snapped frozen hearts from 2-, 6-, and 9-month-old FKRP and BL6 control mice were collected into tubes with 1 mL of TRIzol and homogenized. Total RNA was isolated and washed and the RNA yield and purity was determined using a NanoDrop 2000 microvolume spectrophotometer (ThermoFisher). cDNA was generated using the high-capacity cDNA reverse transcription kit (Applied Biosystems, cat #4368813). The cDNA was added to TaqMan universal PCR master mix (Applied Biosystems, cat #4304437), and the following TaqMan Gene Expression Assays (Applied Biosystems): Nppa, Nppb, and Fn1. GAPDH was used as the reference gene. Real-type PCR was performed using the CFX384 Touch Real-Time PCR Detection System and associated software (Bio-Rad).

### Statistical analysis

Data is presented as mean ± standard deviation (SD). Normality of each phenotype was tested using both the Shapiro-Wilk normality test and visual inspection of histograms except for percent central nucleation and fiber diameter size as there are only three total samples. All tested phenotypes were normally distributed except inflammation in quadriceps of 1- and 9-month-old *BL6*, and 6 months old *P448Lneo−* excised mice, inflammation in gastrocnemius of 2-, 6-, and 9-month-old *BL6*, and 6-month-old *P448Lneo−* excised mice, and inflammation in Triceps of 2-, 6-, and 9-month-old *BL6* mice. For normally distributed parameters, comparisons were made among 6 –month-old *BL6, P448Lneo−*, and *P448Lneo−* excised mice using analysis of the variance (ANOVA) followed by Tukey multiple comparison analysis. A single *t* test was used to compare the *BL6* control group to the *P448Lneo−* group. RT-PCR data were normalized to the 2-month-old *BL6* control group and are presented as fold change. For abnormally distributed parameters, comparisons were made among 6-month-old *BL6, P448Lneo−*, and *P448Lneo−* excised mice using Kruskal-Wallis test followed by Dunn’s multiple comparison analysis and a two-tailed Mann-Whitney test was used to compare the *BL6* control group to the *P448Lneo−* group. A value of *p* < 0.05 was considered statistically significant.

## Results

### Body, organ, and muscle weights

No significant differences were seen in total body weight between *BL6 (control)* and *P448Lneo−* mice (Table 2). There was a significant difference in brain weight normalized to body weight at 6 months of age (*p* < 0.05). The *P448Lneo−* heart showed significantly increased mass when normalized to body weight compared to *BL6* at 9 months of age (*p* < 0.05). The skeletal muscles gastrocnemius, soleus, tibialis anterior, quadriceps, and the triceps from mutant mice all demonstrated significantly increased mass normalized to body weight compared to *BL6* at 6 and 9 months of age (Table 2). *P448Lneo−* mice exercised on the treadmill showed an increase in normalized muscle weight compared to controls for the soleus, tibialis anterior, triceps, and quadriceps muscles (*p* < 0.05; Table 3). Exercised *P448Lneo−* mice also demonstrated a higher normalized muscle weight for the triceps compared to unexercised *P448Lneo−* mice (*p* < 0.05).

**Table 2:** Body, muscle, and organ weights normalized by body weight in P448Lneo*−* (FKRP) and control (BL6) mice showing significant differences at 6 and 9 months of age

Weight (*n* = 8)	1 month		2 months		6 months		9 months	
	BL6	FKRP	BL6	FKRP	BL6	FKRP	BL6	FKRP
Body (g)	19 ± 1.2	19.6 ± 0.9	21.9 ± 1.5	22.3 ± 0.83	28 ± 1.7	30 ± 1.2	33 ± 6.0	31 ± 1.7
GAS/BW(10^−6^)	6.2 ± 0.5	6.3 ± 0.3	6.4 ± 0.4	6.5 ± 0.4	5.4 ± 1	6.0 ± 0.2***	5.0 ± 0.2	6.3 ± 0.5***
Sol/BW(10^−7^)	3.7 ± 0.3	3.7 ± 0.2	3.8 ± 0.9	3.6 ± 0.5	3.0 ± 0.4	3.5 ± 0.2*	3.3 ± 0.3	4.1 ± 0.4**
TA/BW(10^−6^)	2.4 ± 0.4	2.3 ± 0.2	2.3 ± 0.1	2.3 ± 0.2	1.8 ± 0.2	2.2 ± 0.2***	1.4 ± 0.2	2.0 ± 0.3***
Triceps/BW(10^−6^)	4.1 ± 0.4	4.4 ± 0.4	4.4 ± 0.6	4.3 ± 0.4	3.4 ± 0.6	4.5 ± 0.3***	3.3 ± 0.6	5.4 ± 0.9***
Quad/BW(10^−6^)	7.0 ± 0.2	7.3 ± 1.1	5.7 ± 0.8	6.0 ± 0.8	5.3 ± 0.8	6.5 ± 0.9*	5.0 ± 0.9	6.3 ± 0.4**
Heart/BW(10^−6^)	5.0 ± 0.2	5.3 ± 0.5	5.1 ± 0.2	5.2 ± 0.4	4.3 ± 0.3	4.4 ± 0.2	3.9 ± 0.5	4.4 ± 0.4*
Brain/BW(10^−6^)	21 ± 1.2	20 ± 3.6	20 ± 1.4	19 ± 1	16 ± 1.2	15 ± 0.5*	14.5 ± 2.8	14.2 ± 0.1

*p < 0.05; **p < 0.01; ***p < 0.001; ****p < 0.0001; using *t* test when compared to BL6 control mice at same age. Data presented as mean ± SD. *GAS* gastrocnemius, *Sol* soleus, *TA* tibialis anterior, *Quad* quadriceps, *BW* body weight

**Table 3:** Body, muscle, and organ weights normalized by body weights among groups of P448Lneo*−* (FKRP) without and with treadmill exercise (FKRP-treadmill) and control (BL6) mice at 6 months of age

Measurement	BL6 control		FKRP		FKRP-treadmill		Significantly different groups (adjusted *p* values using ANOVA followed by post hoc analysis)
	*N*	Mean ± SD	*N*	Mean ± SD	*N*	Mean ± SD	
Body (g)	8	28 ± 1.7	8	30 ± 1.2	12	29 ± 1.8	NS
GAS/BW(10^−6^)	8	5.4 ± 0.3	8	6.0 ± 0.2	12	6.4 ± 0.7	BL6 vs FKRP: *p* < 0.05, BL6 vs FKRP-treadmill: *p* < 0.001
Sol/BW(10^−7^)	8	3.0 ± 0.4	8	3.5 ± 0.2	12	3.4 ± 0.3	BL6 vs FKRP: *p* < 0.05, BL6 vs FKRP-treadmill: *p* < 0.05.
TA/BW(10^−6^)	8	1.8 ± 0.2	8	2.2 ± 0.2	12	2.4 ± 0.3	BL6 vs FKRP: *p* < 0.01, BL6 vs FKRP-treadmill: *p* < 0.001
Triceps/BW(10^−6^)	8	3.4 ± 0.6	8	4.5 ± 0.3	12	5.2 ± 0.7	BL6 vs FKRP: *p* < 0.01, BL6 vs FKRP-treadmill: *p* < 0.0001 FKRP vs FKRP-treadmill: *p* < 0.05
Quad/BW(10^−6^)	8	5.3 ± 0.8	8	6.5 ± 0.9	12	7.0 ± 1.3	BL6 vs FKRP: *p* < 0.05, BL6 vs FKRP-treadmill: *p* < 0.01
Heart/BW(10^−6^)	8	4.3 ± 0.3	8	4.4 ± 0.2	12	4.4 ± 0.5	NS
Brain/BW(10^−6^)	8	16 ± 1.2	8	15 ± 0.5	12	16 ± 1.2	NS

*GAS* gastrocnemius, *Sol* soleus, *TA* tibialis anterior, *Quad* quadriceps, *BW* body weight, *NS* not significant

## Skeletal muscle

### Grip strength

**Fig. 1:**
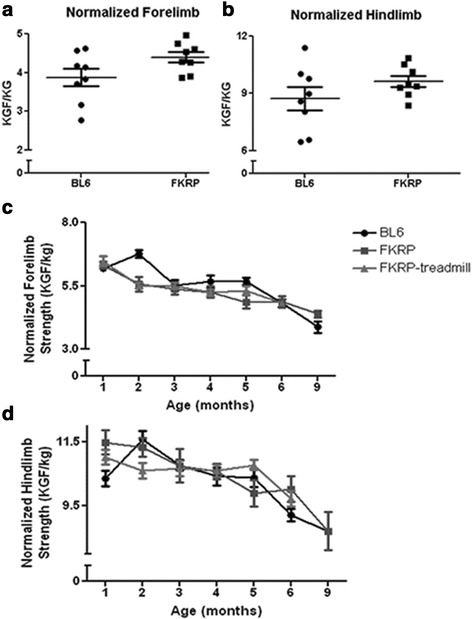
Normalized grip strengths for *P448Lneo−* and control mice. Panel **a** shows no significant differences in normalized forelimb grip strength (kilogram force per kilogram; KGF/kg) at 9 months of age in *P448Lneo−* (FKRP) and control (*BL6*) mice. Panel **b** shows no significant differences in normalized hindlimb grip strength (kilogram force per kilogram; KGF/kg) at 9 months of age in FKRP and control mice. Panel **c** shows normalized forelimb and panel **d** shows normalized hindlimb grip strengths from 1 to 9 months of age for FKRP mice, exercised FKRP mice (FKRP-treadmill; 1–6 months only), and control mice with no significant differences

No significant differences in normalized forelimb or hindlimb grip strength were seen at 9 months of age between *control* and *P448Lneo−* mice (Fig. 1). At 6 months of age, exercise had no significant effects on normalized forelimb or hindlimb grip strength (Fig. 1).

### Activity monitor

There were no significant differences between *BL6* and *P448Lneo−* in horizontal and vertical activity, movement time, rest time, and total distance. While exercised mice showed decreased activity compared to unexercised *P448Lneo−* and *BL6* mice, the differences were not significant (Additional file 1: Figure S1).

### Inflammation

**Fig. 2:**
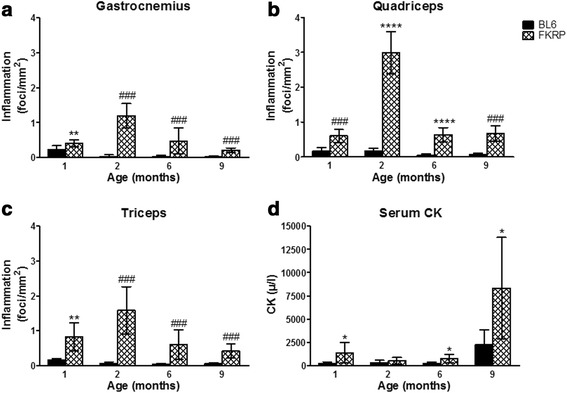
Inflammation levels (foci/mm2) in *P448Lneo−* (FKRP) mice gastrocnemius (panel **a**), quadriceps (panel **b**), and triceps (panel **c**) at 1, 2, 6, and 9 months of age compared to controls (BL6) and serum creatinine kinase (CK) at 9 months of age (panel **d**). Significant increases in inflammation are seen with a peak at 2 months of age. Serum CK levels are significantly increased at 1, 6, and 9 months. Data presented as mean ± standard deviation;*p < 0.05; **p < 0.01; ***p < 0.001; ****p < 0.0001 using *t* test when compared to BL6 control mice at same age; ###*p* < 0.001 using two-tailed Mann-Whitney nonparametric test when compared to BL6 control mice at same age

**Fig. 3:**
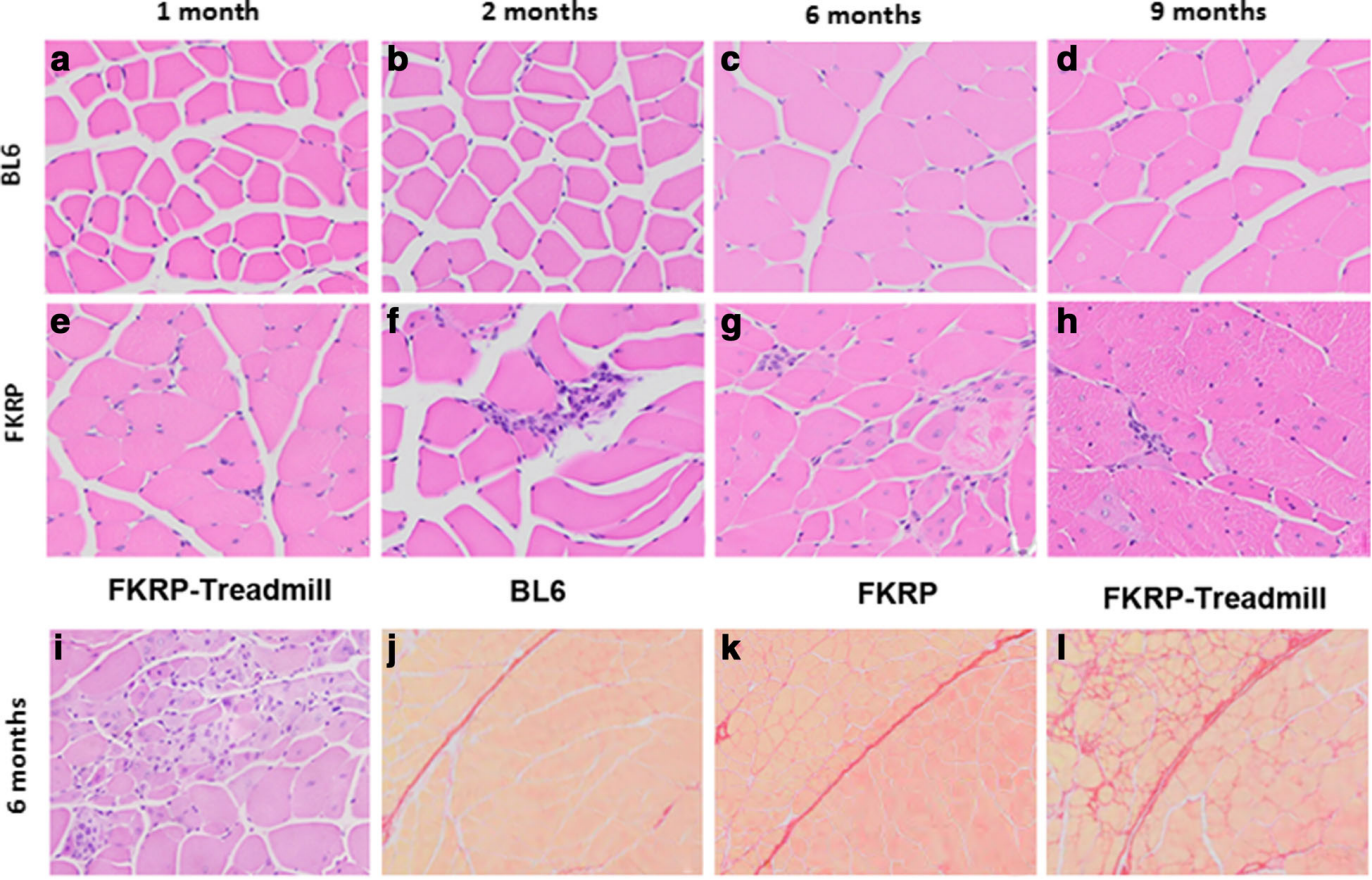
Histology images showing inflammation (hematoxylin and eosin staining at 20x) and fibrosis (picrosirius red staining at 10x) in the quadriceps of *P448Lneo−* (FKRP), exercised *P448Lneo−* (FKRP-treadmill), and control (*BL6*) mice. Panels **a**–**d** show inflammation in *BL6* mice at 1, 2, 6, and 9 months of age. Panels **e**–**h** show inflammation in FKRP mice at 1, 2, 6, and 9 months of age. Panel **i** shows inflammation in FKRP-treadmill mice at 6 months of age. Panels **j**–**l** show fibrosis in BL6, FKRP, and FKRP-treadmill at 6 months of age

Analysis of the skeletal muscle including the gastrocnemius, quadriceps and triceps showed an increase in inflammatory foci at 1 month of age in *P448Lneo−* mice compared to control mice (Figs. 2 and 3). This difference increases by 2 to 4 fold to a maximum inflammation at 2 months of age. The maximum amount of inflammation was noted in the quadriceps muscle. The inflammatory infiltrates then decreased at both 6 and 9 months in all 3 muscles. In *P448Lneo−* mice exercised until 6 months of age, the inflammatory infiltrates increase from 1.8 to 2.2 folds compared to unexercised *P448Lneo−* mice (Table 4; Fig. 3).

**Table 4:** Histological analyses for skeletal muscles and serum creatinine kinase levels among groups of P448Lneo*−* (FKRP) without and with treadmill exercise (FKRP-treadmill) and control (BL6) mice at 6 months of age

Measurement		BL6 control		FKRP		FKRP-treadmill		Significantly different groups (adjusted *p* values using ANOVA followed by post hoc analysis)
		*N*	Mean ± SD	*N*	Mean ± SD	N	Mean ± SD	
Inflammation (foci/mm^2^)	GAS	8	0.03 ± 0.03	8	0.5 ± 0.4	12	0.9 ± 0.3	#BL6 vs FKRP-treadmill: *p* < 0.0001
	Quad	8	0.05 ± 0.04	8	0.6 ± 0.2	12	1.1 ± 0.5	#BL6 vs FKRP: *p* < 0.01, #BL6 vs FKRP-treadmill: *p* < 0.0001
	Triceps	8	0.04 ± 0.06	8	0.6 ± 0.4	12	1.1 ± 0.3	#BL6 vs FKRP: *p* < 0.01, #BL6 vs FKRP-treadmill: *p* < 0.0001
% fibrosis	Quad	8	0.29 ± 0.07	8	0.41 ± 0.18	12	0.61 ± 0.15	BL6 vs FKRP-treadmill: *p* < 0.001, FKRP vs FKRP-treadmill: *p* < 0.05
	GAS	3	0.47 ± 0.23	3	46.18 ± 5.4	3	38.44 ± 5.1	NP
% central nucleation	Quad	3	0.2 ± 0.3	3	56.0 ± 1.9	3	51.1 ± 2.1	NP
	Triceps	3	1.44 ± 1.11	3	65.71 ± 1.1	3	75.16 ± 8.4	NP
	GAS	3	34.27 ± 0.7	3	37.9 ± 3.6	3	42.7 ± 2.6	NP
Fiber diameter size (μm)	Quad	3	48.2 ± 5.5	3	44.8 ± 1.2	3	49.7 ± 1.6	NP
	Triceps	3	37.75 ± 1.2	3	37.03 ± 1.8	3	43.70 ± 2.0	NP
	GAS	3	0.5 ± 0.2	3	7.6 ± 5.4	3	10.7 ± 5.1	NP
SD of % central nucleation	Quad	3	0.4 ± 0.5	3	8.9 ± 3.9	3	12.6 ± 4.4	NP
	Triceps	3	1.28 ± 1.1	3	15.94 ± 1.1	3	8.17 ± 8.4	NP
	GAS	3	1.93 ± 0.7	3	8.5 ± 3.6	3	8.73 ± 2.6	NP
SD of fiber size	Quad	3	11.8 ± 1.3	3	20.5 ± 1.8	3	21.5 ± 1.9	NP
	Triceps	3	1.93 ± 1.2	3	4.8 ± 1.8	3	6.96 ± 2.0	NP
Serum creatinine kinase(μ/l)		8	254 ± 131	7	749 ± 405	12	947 ± 575	BL6 VS. FKRP-treadmill: *p* < 0.05

^#^Kruskal-Wallis test followed by Dunn’s multiple comparison test used. Statistical measures not performed on measures with *N* = 3. *GAS* gastrocnemius, *Quad* quadriceps, *SD* standard deviation, *NP* not performed

### Fibrosis

No significant differences in percent fibrosis in the quadriceps or triceps between *P448Lneo−* and controls were seen at 1, 2, 6, or 9 months of age (Additional file 2: Table S1). *P448Lneo−* exercised mice showed significantly increased percent fibrosis in the quadriceps muscles at 6 months of age compared to unexercised *P448Lneo−* mice (*p* < 0.05) and controls (*p* < 0.001; Table 4; Fig. 3). There were no significant differences in the gastrocnemius and triceps between the 2 mice groups (data not shown).

### Percent central nucleation

Control *BL6* mice showed between 0.2 and 1.4% central nucleation in the quadriceps, gastrocnemius, and triceps from 1 to 9 months. *P448Lneo−* mice showed percent central nucleation of 9.6% at 1 month, 56% at 6 months, and 60.4% at 9 months (Table 4; Additional file 2: Table S1). The percent central nucleation in the quadriceps and triceps were increased in 6-month-old exercised *P448Lneo−* mice compared to unexercised mice while the quadriceps decreased slightly (Table 4). There were differences in the variation (SD of percent central nucleation for each mouse) of *P448Lneo−* and control mice (Additional file 2: Table S1) and *P448Lneo−* exercised mice showed increased SD of percent central nucleation in the triceps compared to controls at 6 months of age (Table 4).

### Fiber diameter

**Fig. 4:**
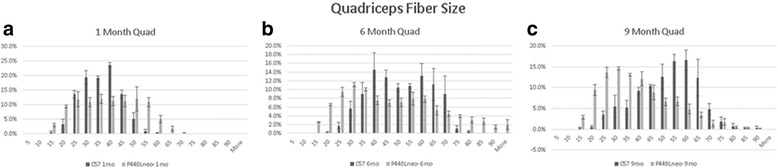
Percent number of muscle fiber diameter sizes (μm) in the quadriceps muscle in *P448Lneo−* and control (*C57*) mice at 1(panel **a**), 6 (panel **b**), and 9 (panel **c**) months of age. Error bars indicate standard deviation

Figure 4 shows the fiber diameters of the quadriceps muscles for *P448Lneo−* mice and controls at 1, 6, and 9 months of age. There was no difference in the average fiber size of *P448Lneo−* and control *BL6* mice at 1 and 6 months of age. At 9 months of age, *P448Lneo−* mice have smaller average fiber diameter compared to control *BL6* (Additional file 2: Table S1). There was a greater variation in fiber sizes (SD of fiber size for each mouse) in *P448Lneo−* mice at 1 and 6 months of age compared to control *BL6* (Additional file 2: Table S1). There were no differences in fiber size, but unexercised and exercised P448Lneo*−* mice showed increased SD of fiber size for each mouse compared to controls in the quadriceps, gastrocnemius, and triceps (Table 4).

## Serum creatinine kinase (CK)

Serum CK was significantly increased in *P448Lneo−* mice compared to control *BL6* at 1, 6, and 9 months of age (*p* < 0.05; Fig. 2). At 6 months of age, exercised *P448Lneo−* mice showed significantly increased serum CK levels compared to BL6 controls (*p* < 0.05; Table 4).

## Respiratory muscle

### Plethysmography

**Fig. 5:**
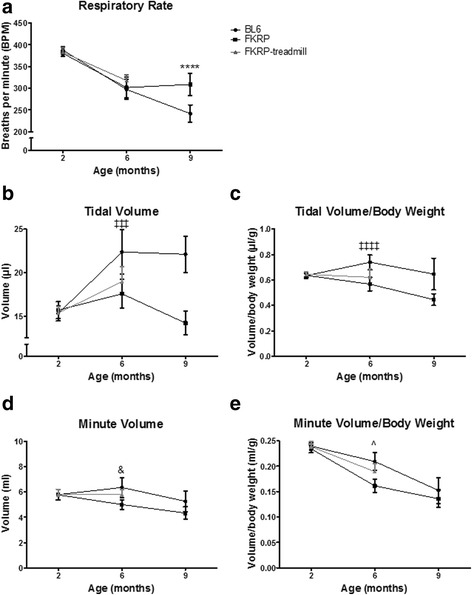
Plethysmography results in *P448Lneo−* (FKRP) mice, exercised *P448Lneo−* mice (FKRP-treadmill), and controls (*BL6*) at 2, 6, and 9 months of age. Respiratory rates (panel **a**) are significantly less in control mice compared to FKRP. Tidal volume (panel **b**) and normalized tidal volume (panel **c**) are significantly increased in controls. Minute volume (panel **d**) and normalized minute volume (panel **e**) show significant changes only at 6 months. FKRP-treadmill mice only measured at 2 and 6 months. ****p < 0.0001 between *BL6* and FKRP; ‡‡‡*p* < 0.001, ‡‡‡‡*p* < 0.0001, and *p* < 0.001 between *BL6* and FKRP/FKRP-treadmill; ^*p* < 0.05 among all groups. Data presented as mean ± standard deviation

*P448Lneo−* mice demonstrated a reduced decline in respiratory rate over time compared to *BL6* controls at 9 months of age (*p* < 0.001; Fig. 5). *P448Lneo−* mice also showed significantly decreased tidal volumes (*p* < 0.001), normalized tidal volumes (*p* < 0.01), and minute volumes (*p* < 0.001) compared to *BL6* controls at 6 and 9 months of age. There were significant differences in plethysmography measures at 6 months that were improved in exercised *P448Lneo−* mice compared to unexercised *P448Lneo−* including tidal volume (*p* < 0.001), minute volume (*p* < 0.01), and normalized minute volume (*p* < 0.01; Fig. 5).

### Inflammation

**Fig. 6:**
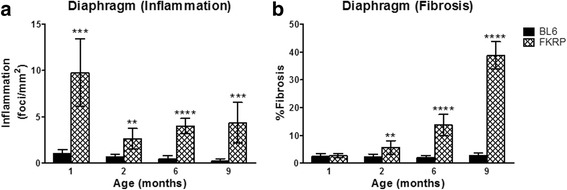
Significantly increased diaphragm inflammation (panel **a**) and fibrosis (panel **b**) are seen in *P448Lneo−* mice (FKRP) compared to controls (*BL6*) at 2, 6, and 9 months of age. *p < 0.05; **p < 0.01; ***p < 0.001; ****p < 0.0001 compared to BL6 control at same age. Data presented as mean ± standard deviation

**Fig. 7:**
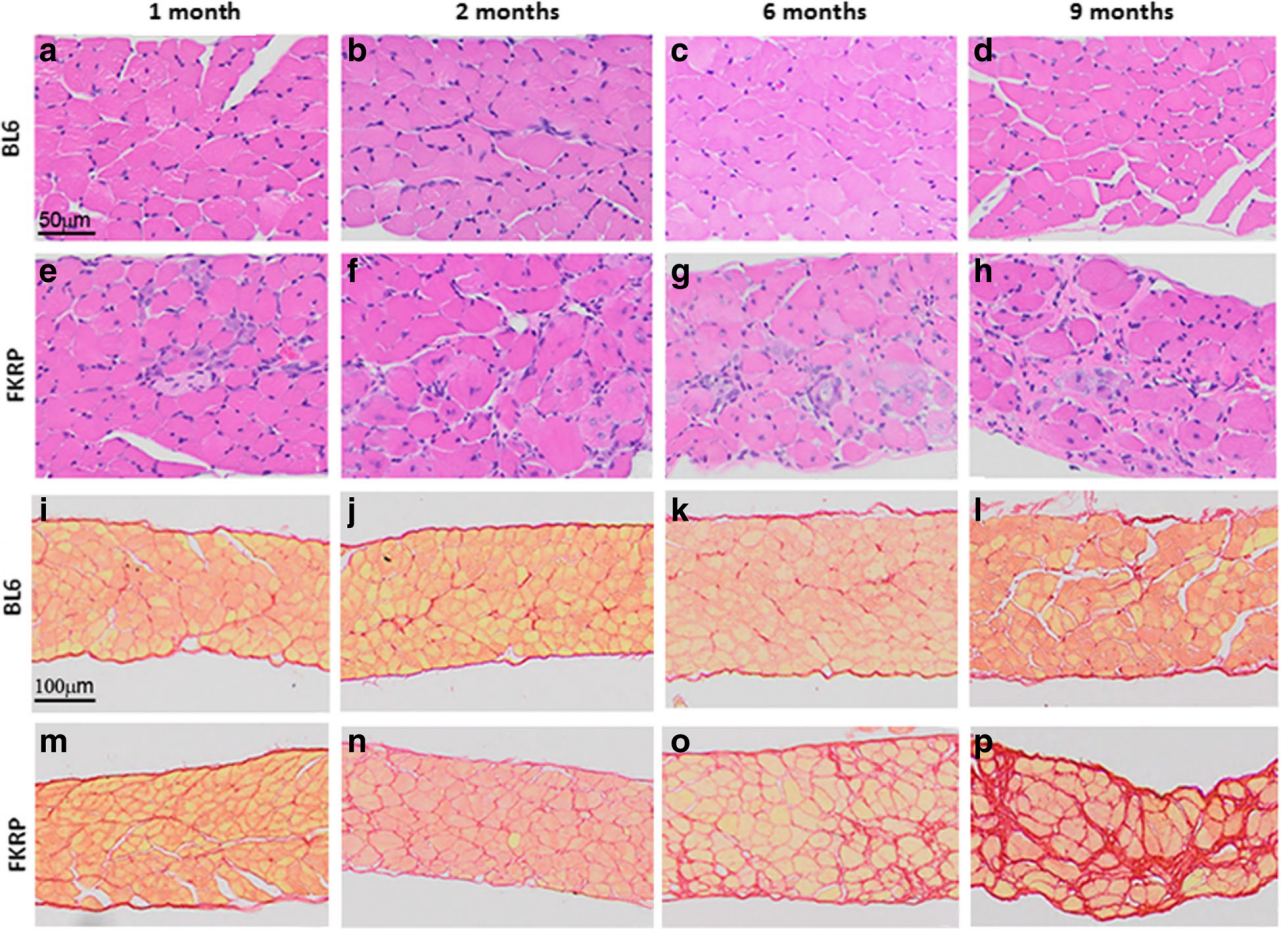
Inflammation (hematoxylin and eosin staining at 20x) and fibrosis (picrosirius red staining at 10x) in diaphragm in *P448Lneo−* (FKRP) and control (*BL6*) mice. Panels **a**–**d** show inflammation in the diaphragm of *BL6* mice at 1, 2, 6, and 9 months of age. Panels **e**–**h** show inflammation in the diaphragm of FKRP mice at 1, 2, 6, and 9 months of age. Panels **i**–**l** show fibrosis in the diaphragm of *BL6* mice at 1, 2, 6, and 9 months of age. Panels **m**–**p** shows fibrosis in the diaphragm of FKRP mice at 1, 2, 6, and 9 months of age

The diaphragm of *P448Lneo−* mice showed the most inflammatory infiltrates at 1 month of age (*p* < 0.001; Figs. 6 and 7). The infiltrates decreased but remained significant compared to controls from 2 to 9 months of age (*p* < 0.0.01). Exercised *P448Lneo−* mice showed significant inflammation that was increased compared to unexercised *P448Lneo−* mice and *BL6* controls at 6 months of age (*p* < 0.05; Table 5).

**Table 5:** Histological analyses of the diaphragm among groups of *P448Lneo−* (FKRP) with and without treadmill exercise (FKRP-treadmill) and control (*BL6*) mice at 6 months of age

Measurement	BL6 control		FKRP		FKRP-treadmill		Significantly different groups (adjusted *p* values using ANOVA followed by post hoc analysis)
	*N*	Mean ± SD	*N*	Mean ± SD	*N*	Mean ± SD	
Inflammation (foci/mm^2^)	8	0.5 ± 0.3	8	4.0 ± 0.8	12	8.7 ± 2.8	BL6 vs FKRP: *p* < 0.01, BL6 vs FKRP-treadmill: *p* < 0.0001 FKRP vs FKRP-treadmill: *p* < 0.0001
% fibrosis	8	2.0 ± 0.7	8	13.7 ± 3.8	12	13.4 ± 2.2	BL6 vs FKRP: *p* < 0.0001, BL6 vs FKRP-treadmill: *p* < 0.0001
% central nucleation	3	2.4 ± 0.4	3	24.8 ± 2.0	3	44 ± 9.4	NP
Fiber diameter size (μm)	3	24.6 ± 2.6	3	21.3 ± 1.2	3	26.7 ± 2.5	NP
SD of % central nucleation for each mouse	3	1.6 ± 1.2	3	4.9 ± 3.3	3	7.7 ± 2.3	NP
SD of fiber size for each mouse	3	4.5 ± 0.8	3	6.3 ± 0.2	3	8.1 ± 0.2	NP

Statistical analyss not performed on measures with *N* = 3. *SD* standard deviation, *NP* not performed

### Percent central nucleation diaphragm

Control *BL6* mice showed between 2.3 and 3.7% central nucleation in the diaphragm from 1 to 9 months of age. 1-month-old *P448Lneo−* mice showed 4.4% central nucleation, which increased to 34% at 9 months old (Additional file 3: Table S2). 6-month-old unexercised *P448Lneo−* mice showed 25% central nucleation, and this increased to 44% in exercised mice (Table 5). There is no difference in the variation (SD of percent central nucleation for each mouse) among *P448Lneo−* exercised, unexercised, and control mice (Table 5; Additional file 3: Table S2).

### Diaphragm fiber diameter

**Fig. 8:**
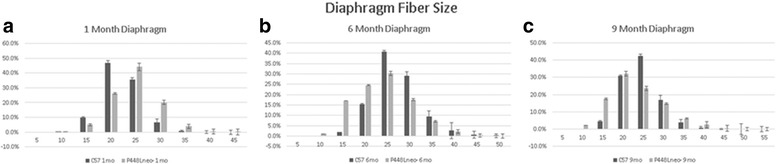
Percent number of muscle fiber diameter sizes (μm) in the diaphragm muscle in *P448Lneo−* and control (*C57*) mice at 1(panel **a**), 6 (panel **b**), and 9 (panel **c**) months of age. Error bars indicate standard deviation

Figure 8 shows the fiber diameters of the diaphragm muscle for *P448Lneo−* mice and *BL6* controls at 1, 6, and 9 months of age. No differences were seen in average fiber size in the mutant mice compared to controls in the diaphragm, but there was greater variation in fiber sizes (SD of fiber size for each mouse) in *P448Lneo−* mice at 1 and 6 months of age compared to controls (Additional file 3: Table S2) and between unexercised and exercised *P448Lneo−* mice at 6 months of age (Table 5).

### Diaphragm fibrosis

There were no significant differences in percent fibrosis of the diaphragm at 1 month of age; however, there was significantly increased percent fibrosis in the diaphragms of 2-, 6- (unexercised and exercised), and 9-month-old *P448Lneo−* mice compared to controls (*p* < 0.01; Table 5; Fig. 6).

## Cardiac muscle

### Echocardiography

**Fig. 9:**
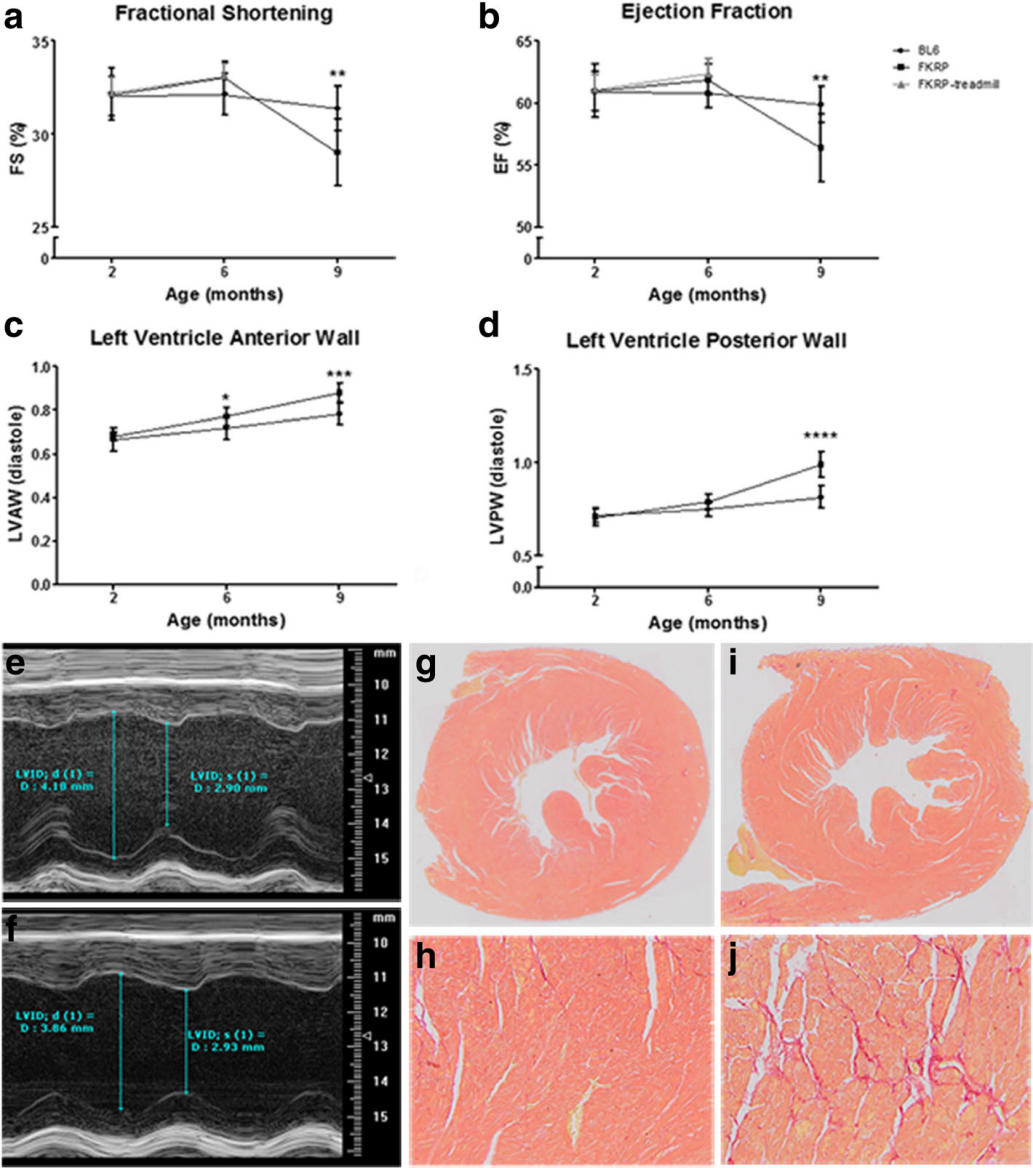
Cardiac phenotypes in *P448Lneo−* (FKRP) and control (BL6) mice. At 9 months of age, there was significantly decreased systolic function measured via fractional shortening percent (FS%; panel **a**) and ejection fraction (EF%; panel **b**) in *P448Lneo−* mice compared to controls (*p* < 0.01). FKRP-treadmill mice were only measured at 2 and 6 months. FKRP mice showed significantly increased left ventricular anterior wall (LVAW) thickness at 6 and 9 months of age (panel **c**). Left ventricular posterior wall (LVPW) thickness was significantly increased in FKRP mice at 9 months (panel **d**). Panel **e** is an echo image in the parasternal short axis showing the M-mode tracing for a 9-month-old *BL6* control mouse. The left ventricular internal diameter in diastole measured 4.18 mm. Panel **f** is an echo image in the parasternal short axis showing the M-mode image for a 9-month-old FKRP mouse. The left ventricular internal diameter in diastole measured 3.86 mm. FKRP mice showed a smaller left ventricular internal diameter in diastole at 9 months of age. Picrosirius red staining of the left ventricle (panel **g** 10x; panel **h** 20x) of a control mouse at 9 months of age shows no significant collagen staining. Picrosirius red staining of the left ventricle (panel **i** 10x; panel **j** 20x) of a FKRP mouse at 9 months of age shows patchy, diffuse collagen staining. There was significantly increased cardiac fibrosis in 9-month-old FKRP mice compared to controls

Echocardiographic data collected at 2 and 6 months were not significantly different between *P448Lneo−* mice and controls except for heart rates (Additional file 4: Table S3). At 9 months of age, there was significantly decreased systolic function measured via SF in the mutant mice (29 ± 2%) compared to controls (31 ± 1%; *p* < 0.01; Fig. 9). The left ventricular internal diameter in diastole measured in the parasternal short axis was smaller in *P448Lneo−* mice compared to controls and corresponded to smaller left ventricular volume in diastole and a significantly decreased left ventricular stroke volume (*p* < 0.01). The myocardial thickness of the left ventricular infero-posterior wall was significantly increased in *P448Lneo−* mice compared to controls (*p* < 0.0001), and this corresponded with a significantly increased left ventricular mass in the mutant mice (*p* < 0.001; Fig. 9; Additional file 4: Table S3). The myocardial performance index (MPI) was also noted to be significantly increased in *P448Lneo−* mice compared to controls at 9 months of age. This was related to a significantly decreased isovolumic relaxation time (IVRT) seen in the mutant mice (12.1 ms versus 15.3 ms in controls; *p* < 0.04). This increase may be related to decreased ventricular compliance. Heart rates at 6 months of exercised *P448Lneo−* mice (463 ± 40 beats per minute; BPM) and unexercised *P448Lneo−* (461 ± 27 BPM) were significantly increased compared to controls (433 ± 29 BPM; *p* < 0.05).

### Myocardial fibrosis

There were no significant differences in myocardial fibrosis at 1, 2, and 6 months of age between *P448Lneo−* mice compared to controls. There was significantly increased myocardial percent fibrosis at 9 months of age in *P448Lneo−* mice (0.69 ± 0.24) compared to controls (0.38 ± 0.17; *p* < 0.05; Fig. 9).

### Myocardial mRNA expression

**Fig. 10:**
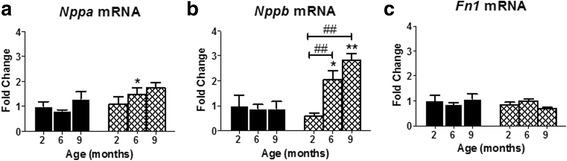
Real-time PCR of *Nppa* (panel **a**), *Nppb* (panel **b**), and *Fn1* (panel **c**) for *P448Lneo−* (FKRP) and control (*BL6*) mice at 2, 6, and 9 months of age. Fold-changes are shown relative to 2-month-old control mice. Data are presented as mean and error bars denote SD for *n* = 4–6 per group. * represents a significant difference between age-matched FKRP and BL6 mice, # represents a significant difference across age for FKRP mice

mRNA expression for natriuretic peptide type A (*Nppa*) was significantly increased in *P448Lneo−* mice compared to controls at 6 months (*p* < 0.05) and for natriuretic peptide type B (*Nppb*) at 6 (*p* < 0.05) and 9 months of age (*p* < 0.01; Fig. 10). There were no differences in mRNA expression of fibronectin 1 (*Fn1*) between *P448Lneo−* and control mice at all ages (Fig. 10).

## Discussion

In this study, we further phenotyped the *P448Lneo−* mouse model of FKRP-related limb girdle muscular dystrophy. One important aspect of this study is to better understand and validate cardiac muscle disease in the *FKRP* mutant mouse. Earlier studies reported mild effects of the disease on the histology and functions of the cardiac muscle. Blaeser et al. [19] reported an EF 49 ± 5% in *P448Lneo−* and 55 ± 9% in *BL6* control mice at 10 months of age, although this difference was not statistically significant. However, Blaeser et al. did find a significant difference in EF between *P448Lneo−* (55 ± 5%) and *BL6* (62 ± 7%) at the age of 6 months [19]. Maricelli et al. (2017) also demonstrated decreased EF and SF at 6 months of age in male and female *P448Lneo−* mice compared to controls, with female mice demonstrating more significant deficits [24]. In this current study, we demonstrated significant decrease in systolic cardiac function in *P448Lneo−* male mice compared to *BL6* at 9 months of age. *P448Lneo−* mice had a SF of 29% compared to 31% in controls (*p* < 0.01). This corresponds to an EF of 56% in *P448Lneo−* mice compared to 60% in controls. However, we show no significant differences in cardiac function at 6 months of age. We also demonstrated increased myocardial wall thickness and left ventricular mass at 9 months of age in *P448Lneo−* mice associated with increased mRNA expression of Nppa and Nppb [28].

Histopathology demonstrated an increase in myocardial fibrosis. Blaeser et al. showed patchy myocardial fibrosis that was 4% of measured area at 6 months of age and increased to about 6% at 12 months of age, compared to approximately 1% in *BL6* controls [19]. The current study also demonstrated an increase in myocardial fibrosis and showed approximately twice the amount of fibrosis in *P448Lneo−* mice compared to controls at 9 months of age. The increasing myocardial fibrosis with age likely leads to worsening systolic function as these mice get older. Data from all studies are therefore consistent indicating that lack of functional glycosylation of a-DG results in a mild but progressive degeneration and fibrosis in the cardiac muscle. This leads to a clear trend of decrease in cardiac systolic function. However, demonstration of significance in cardiac function between normal and mutant mice is dependent on age, method of detection, and likely requires a larger cohort size.

Respiratory disease is seen in the clinical spectrum of FKRP-mediated LGMD [29]. This was also demonstrated in the *P448Lneo−* mouse model. Blaeser et al. demonstrated significant pathology in the diaphragm starting at 6 weeks of age. By 6 months of age, there were large areas of inflammatory infiltration. By 10 and 12 months of age, the area of fibrotic tissue increased to approximately 60% with the majority of fibers demonstrating central nucleation [19]. An earlier study also showed severe pathology in the diaphragm with clear variation in fiber size, the presence of necrotic fibers and central nucleation (17.6%) [16]. Maricelli et al. also showed changes in central nucleation of the diaphragm at 3 months of age [24]. The current study confirms that the decreased normalized tidal and minute volumes at 6 and 9 months of age correspond with increased inflammation and fibrosis in the diaphragm. We also show a functional decline in respiration with age. Interestingly, *P448Lneo−* mice demonstrated a reduced decline in respiratory rate over time. This is likely related to the fact that older mice have reduced tidal volumes, and they can maintain higher respiratory rates for their activity due to the compensatory effort by the remaining muscles. However, respiratory rates in more severe dystrophic phenotypes, and perhaps also patients lacking regeneration capacity, will likely decrease more significantly with age. Interestingly, exercised *P448Lneo−* mice showed less tidal and minute volume loss compared to unexercised mice. This may be again related to exercise-related compensatory regeneration in the diaphragm indicated by significant increase in central nucleation (44%) compared to unexercised mice (25%). Other potential factors, not evaluated in this study, including pulmonary inflammation and vascular function could also be involved. Exercised mice showed significantly increased diaphragm muscle inflammation compared to unexercised mice. This could lead to a more dramatic decrease in respiratory function at an older age; further studies are needed. The functional parameters of plethysmography and associated pathologic changes in the diaphragm make the *P448Lneo−* a strong model for respiratory disease in *FKRP*-related LGMD.

We did not demonstrate any significant functional differences in muscle strength or activity in unexercised *P448Lneo−* mice compared to controls. This is likely related to the significant evidence of skeletal muscle regeneration present in the mouse model. Blaeser et al. showed that all limb skeletal muscles had severe degeneration (necrotic fibers) and regeneration (central nucleation) as a predominant feature with relatively limited fibrosis [19]. Cycles of muscle degeneration and regeneration were clearly indicated by the significant variation in fiber size and central nucleation in more than 37% of the muscle fibers [16]. We also demonstrated increased regeneration by percent central nucleation in the quadriceps of *P448Lneo−* mice at 2, 6 (both unexercised and exercised), and 9 months of age. Interestingly, fibrosis was limited in skeletal muscle (quadriceps and triceps) of the unexercised mice, but was significantly increased in exercised mice. Maricelli et al. used a modified exercise protocol, based on studies from Rocco et al. [30], which included two sessions where mice exercised to exhaustion. This protocol elicited both functional and histological change in exercised mice including decreased grip strength, short time to exhaustion, increased fibrosis in the diaphragm, and increased serum CK levels of *P448Lneo−* mice compared to unexercised mice and controls [24, 30]. While an optimal exercise protocol is not yet known, degree of exercise is clearly important to the course of disease progression, and the *P448Lneo−* mice provide a model for such further analysis.

## Conclusions

This study provides more comprehensive outcome measures for the *P448Lneo−* mouse model of FKRP deficiency. The study shows significant decrease in cardiac function at 9 months of age. This study is the first to provide respiratory function data demonstrating significantly decreased tidal and minute volumes in the mouse model at 6 and 9 months of age. A chronic exercise protocol demonstrated increased skeletal muscle fibrosis, but improved respiratory function at 6 months of age in mutant mice. Further studies are needed to better understand the complexities of exercise on muscle pathology and disease progression. The results provide new data on outcome measures for future preclinical drug trials using the *P448Lneo−* mouse as a model system for FKRP deficiency muscular dystrophy.

## Additional files

Additional file 1: **Figure S1.** Behavioral activity monitoring in *P448Lneo−* (FKRP), exercised *P448Lneo−* (FKRP-treadmill), and control (*BL6*) mice from 1 to 9 months of age. FKRP-treadmill mice were only measured until 6 months of age. Panel A: vertical activity (VACTV) data; panel B: horizontal activity (HACTV) data; panel C: total distance traveled (cm) during session (TOTDIST) data; panel D: time (sec, seconds) spent in movement (MOVTIME); panel E: time (sec, seconds) spent resting (RESTIME). No significant differences were seen between groups for all measures. (TIFF 136 kb)

Additional file 2: **Table S1.** Histological analyses for skeletal muscles and serum creatinine kinase levels in *P448Lneo−* (FKRP) and control (*BL6*) mice at 1, 2, 6, and 9 months of age. (DOCX 16 kb)

Additional file 3: **Table S2.** Histological analyses of the diaphragm in *P448Lneo−* (FKRP) and control (*BL6*) mice at 1, 2, 6, and 9 months of age. (DOCX 15 kb)

Additional file 4: **Table S3.** Echocardiography results for *P448Lneo−* (FKRP) and control (*BL6*) mice at 2, 6, and 9 months of age showing increased cardiac hypertrophy and decreased systolic function at 9 months of age. (DOCX 15 kb)

## Abbreviations

ANOVA: analysis of the variance
BL6: C57BL/6J control mice
bpm: Beats per minute
BPM: Breaths per minute
BW: Body weight
C57: C57BL/6J control mice
cDNA: Complementary deoxyribonucleic acid
CK: Creatinine kinase
CMD: Congenital muscular dystrophies
CO: Cardiac output
DGC: Dystrophin-glycoprotein complex
ECM: Extracellular matrix
EF: Ejection fraction
FKRP: Fukutin-related protein
Fn1: Fibronectin 1
GADPH: Glyceraldehyde 3-phosphate dehydrogenase
GAS: Gastrocnemius
HR: Heart rate
IACUC: Institutional Animal Care and Use Committee
LGMD: Limb-girdle muscular dystrophy
LV mass cor: Left ventricular mass corrected
LV: Left ventricular
LVAW, d: Left ventricular anterior wall thickness in diastole
LVID, d: Left ventricular internal dimension in diastole
LVPW, d: Left ventricular posterior wall thickness in diastole
LVVol, d: Left ventricular volume in diastole
MPI: Myocardial performance index
mRNA: Messenger ribonucleic acid
MV: Minute ventilation
NIH: National Institutes of Health
Nppa: Natriuretic peptide type a
Nppb: Natriuretic peptide type b
NS: Not significant
PCR: Polymerase chain reaction
Quad: Quadriceps
RNA: Ribonucleic acid
SD: Standard deviation
SF: Shortening fraction
Sol: Soleus
SV: Stroke volume
TA: Tibialis anterior
Tri: Triceps
TV: Tidal volume
α-DG: Alpha-dystroglycan
