# Supplementary figures and images for: Skeletal, cardiac, and respiratory muscle function and histopathology in the P448Lneo− mouse model of FKRP-deficient muscular dystrophy
Source: Skelet Muscle. 2018 Apr 6;8:13. doi: 10.1186/s13395-018-0158-x (PMC5889611; doi:10.1186/s13395-018-0158-x)

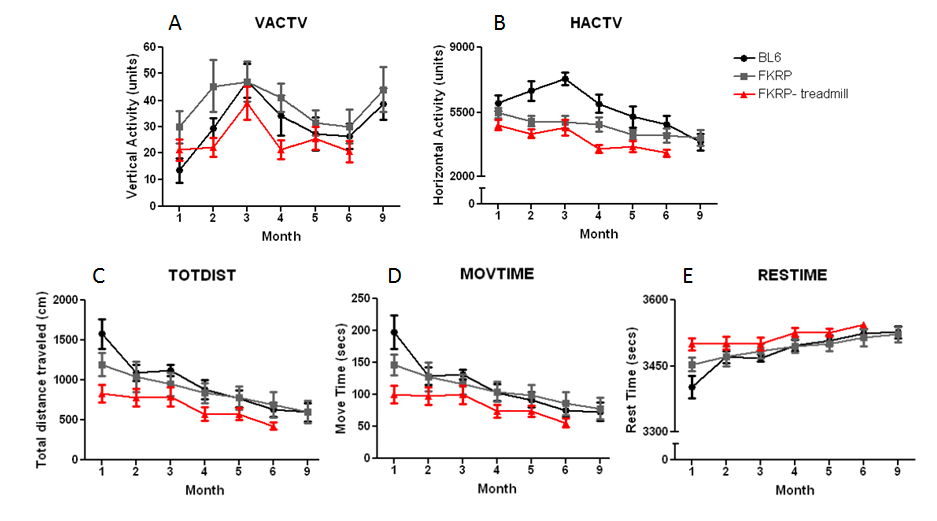

Supplement: Supplementary file 1 — Figure S1. Behavioral activity monitoring in P448Lneo− (FKRP), exercised P448Lneo− (FKRP-treadmill), and control (BL6) mice from 1 to 9 months of age. FKRP-treadmill mice were only measured until 6 months of age. Panel A: vertical activity (VACTV) data; panel B: horizontal activity (HACTV) data; panel C: total distance traveled (cm) during session (TOTDIST) data; panel D: time (sec, seconds) spent in movement (MOVTIME); panel E: time (sec, seconds) spent resting (RESTIME). No significant differences were seen between groups for all measures. (TIFF 136 kb) [file 13395_2018_158_MOESM1_ESM.tif]
