# Supplementary material for: Skeletal, cardiac, and respiratory muscle function and histopathology in the P448Lneo− mouse model of FKRP-deficient muscular dystrophy
Source: Skelet Muscle. 2018 Apr 6;8:13. doi: 10.1186/s13395-018-0158-x (PMC5889611; doi:10.1186/s13395-018-0158-x)
Supplement: Supplementary file 2 — Table S1. Histological analyses for skeletal muscles and serum creatinine kinase levels in P448Lneo− (FKRP) and control (BL6) mice at 1, 2, 6, and 9 months of age. (DOCX 16 kb) [file 13395_2018_158_MOESM2_ESM.docx]

**Additional file 2: Table S1:** Histological analyses for skeletal muscles and serum creatine kinase levels in P448Lneo- (FKRP) and control (BL6) mice at 1, 2, 6 and 9 months of age

| **Measurement/Muscle** | | **1 months** | | **2 months** | | **6 months** | | **9 months** | |
| --- | --- | --- | --- | --- | --- | --- | --- | --- | --- |
|  |  | **BL6** | **FKRP** | **BL6** | **FKRP** | **BL6** | **FKRP** | **BL6** | **FKRP** |
| % Fibrosis  (n=8) | Quad | 0.34±0.06 | 0.34±0.07 | 0.29±0.10 | 0.30±0.05 | 0.29±0.07 | 0.41±0.18 | 0.29±0.06 | 0.35±0.07 |
|  | Tri | 0.31±0.10 | 0.32±0.12 | 0.32±0.05 | 0.33±0.11 | 0.35±0.08 | 0.40±0.07 | 0.36±0.16 | 0.37±0.09 |
| % Central Nucleation (n=3) | Quad | 0.5±0.1 | 9.6±4.3 | - | - | 0.2±0.3 | 56.0±1.9 | 0.5±0.4 | 60.4±6.6 |
| Fiber diameter size (µm) (n=3) | Quad | 33.4±2.5 | 37.2±1.6 | - | - | 48.2±5.5 | 44.8±1.2 | 49.3±3.7 | 36.1±1.7 |
| SD of % Central Nucleation (n=3) | Quad | 0.5±0.4 | 7.4±4.5 | - | - | 0.4±0.5 | 8.9±4 | 0.5±0.4 | 13.1±7.7 |
| SD of fiber size (n=3) | Quad | 7.1±0.2 | 12.5±1.5 | - | - | 11.8±1.3 | 20.5±1.8 | 12.2±1.7 | 14.5±1.9 |

Data presented as mean±SD.

There were no statistically significant differences. Not all measures performed at 2 months of age. Statistical analysis not performed on measures with N=3.

GAS: gastrocnemius; Quad: Quadriceps; Tri: triceps; SD: standard deviation; CK: creatine kinase.
