# Supplementary material for: Skeletal, cardiac, and respiratory muscle function and histopathology in the P448Lneo− mouse model of FKRP-deficient muscular dystrophy
Source: Skelet Muscle. 2018 Apr 6;8:13. doi: 10.1186/s13395-018-0158-x (PMC5889611; doi:10.1186/s13395-018-0158-x)
Supplement: Supplementary file 3 — Table S2. Histological analyses of the diaphragm in P448Lneo− (FKRP) and control (BL6) mice at 1, 2, 6, and 9 months of age. (DOCX 15 kb) [file 13395_2018_158_MOESM3_ESM.docx]

**Additional file 3: Table S2**: Histological analyses of the diaphragm in P448Lneo- (FKRP) and control (BL6) mice at 1, 2, 6 and 9 months of age

| **Measurement** | **1 months** | | **2 months** | | **6 months** | | **9 months** | |
| --- | --- | --- | --- | --- | --- | --- | --- | --- |
|  | **BL6** | **FKRP** | **BL6** | **FKRP** | **BL6** | **FKRP** | **BL6** | **FKRP** |
| % Central Nucleation (n=3) | 3.7±2.6 | 4.4±3.2 | - | - | 2.4±0.4 | 24.8±2 | 2.3±0.3 | 34.1±6.5 |
| Fiber diameter size (µm) (n=3) | 19.5±1.4 | 22.1±1.0 | - | - | 24.6±2.6 | 21.3±1.2 | 21.8±1.2 | 20.6±1.9 |
| SD of % Central Nucleation (n=3) | 1.4±0.8 | 2.3±1.2 | - | - | 1.6±1.2 | 4.9±3.3 | 0.6±0.5 | 6.8±3.6 |
| SD of fiber size (n=3) | 3.6±0.5 | 4.4±0.4 | - | - | 4.5±0.8 | 6.3±0.2 | 4.4±0.4 | 6.4±1.0 |

Data presented as mean±SD.

There were no statistically significant differences. Not all measures performed at 2 months of age. Statistical analysis not performed on measures with N=3.

SD: standard deviation.
