# Supplementary material for: Skeletal, cardiac, and respiratory muscle function and histopathology in the P448Lneo− mouse model of FKRP-deficient muscular dystrophy
Source: Skelet Muscle. 2018 Apr 6;8:13. doi: 10.1186/s13395-018-0158-x (PMC5889611; doi:10.1186/s13395-018-0158-x)
Supplement: Supplementary file 4 — Table S3. Echocardiography results for P448Lneo− (FKRP) and control (BL6) mice at 2, 6, and 9 months of age showing increased cardiac hypertrophy and decreased systolic function at 9 months of age. (DOCX 15 kb) [file 13395_2018_158_MOESM4_ESM.docx]

**Additional file 4: Table S3**: Echocardiography results for P448Lneo- (FKRP) and control (BL6) mice at 2, 6 and 9 months of age showing increased cardiac hypertrophy and decreased systolic function at 9 months of age.

| **Outcome (n=8)** | **2 months** | | **6 months** | | **9 months** | |
| --- | --- | --- | --- | --- | --- | --- |
|  | **BL6** | **FKRP** | **BL6** | **FKRP** | **BL6** | **FKRP** |
| LVID, d (mm) | 3.77±0.22 | 3.85 ± 0.21 | 4.1±0.13 | 4.1 ± 0.16 | 4.2±0.15 | 3.9 ± 0.1 |
| LV Vol, d (ul) | 61±8 | 63 ± 8 | 73±6 | 74 ± 7 | 79±8 | 73 ± 6 |
| SV (ul) | 37±5 | 38 ± 5 | 45±4 | 45 ± 4 | 47±4 | 41 ± 3** |
| LV mass cor (mg) | 70±8 | 74±5 | 86±8 | 94±6 | 103±7 | 115±8*** |
| HR (bpm) | 433±29 | 461 ± 21* | 417±7 | 461 ± 27** | 393±22 | 412 ± 50 |
| CO (ml/minute) | 16±2.7 | 18 ± 2.5 | 19±2 | 21 ± 2 | 19±1.4 | 17 ± 1.6 |
| MPI | 0.5±0.02 | 0.5±0.02 | 0.56±0.03 | 0.56±0.03 | 0.6±0.03 | 0.53±0.06* |

*p<0.05; **p<0.01; ***p<0.001; ****p<0.0001; using T-test when compared to BL6 control mice at same age. Data presented as mean±SD. N = 8 for all groups.

LVID, d – left ventricular internal dimension in diastole; LV Vol, d – left ventricular volume in diastole; SV – stroke volume; LV mass cor– left ventricular mass corrected; HR – heart rate; bpm – beats per minute; CO – cardiac output; MPI – myocardial performance index.
